# Sudden Bilateral Sensorineural Hearing Loss Associated with HLA A1-B8-DR3 Haplotype

**DOI:** 10.1155/2013/590157

**Published:** 2013-09-09

**Authors:** G. Psillas, M. Daniilidis, A. Gerofotis, K. Veros, A. Vasilaki, I. Vital, K. Markou

**Affiliations:** ^1^1st Academic ENT Department, Ahepa University Hospital, Aristotle University, 54006 Thessaloniki, Greece; ^2^Clinical Immunology and Immunogenetics Lab., 1st Department of Internal Medicine, Ahepa University Hospital, Aristotle University, 54006 Thessaloniki, Greece; ^3^1st Department of Internal Medicine, Ahepa University Hospital, Aristotle University, 54006 Thessaloniki, Greece

## Abstract

Sudden sensorineural hearing loss may be present as a symptom in systemic autoimmune diseases or may occur as a primary disorder without another organ involvement (autoimmune inner ear disease). The diagnosis of autoimmune inner ear disease is still predicated on clinical features, and to date specific diagnostic tests are not available. We report a case of bilateral sudden hearing loss, tinnitus, intense rotatory vertigo, and nausea in a female patient in which the clinical manifestations, in addition to raised levels of circulating immune complexes, antithyroglobulin antibodies, and the presence of the HLA A1-B8-DR3 haplotype, allowed us to hypothesize an autoimmune inner ear disease. Cyclosporine-A immunosuppressive treatment in addition to steroids helped in hearing recovery that occurred progressively with normalization of the hearing function after a five-month treatment. Cyclosporine-A could be proposed as a therapeutic option in case of autoimmune inner ear disease allowing the suspension of corticosteroids that, at high dose, expose patients to potentially serious adverse events.

## 1. Introduction

Sudden sensorineural hearing loss (SSNHL) is defined as a loss of 30 dB or more at one or more frequencies over a period of 3 days or less; it is a frustrating and frightening condition, especially if the hearing loss is bilateral. Although vascular and viral mechanisms have been implicated in the aetiology of SSNHL, a high prevalence of autoantibody titer was reported in cases of bilateral SSNHL, possibly suggesting an underlying autoimmune process [1]. We present here a case of bilateral SSNHL associated with the involvement of autoantibodies in the presence of a certain human leukocyte antigen (HLA) haplotype, the HLA A1-B8-DR3 superhaplotype.

## 2. Case Report

A 28-year-old female presented to our clinic complaining of bilateral hearing loss, tinnitus, intense rotatory vertigo, and nausea. She reported that, during the previous two weeks, she experienced 2 episodes of vertigo lasting several hours with associated nausea and vomiting, fullness in her right ear, and pain in both ears. A CT scan and an MRI were normal.

**Figure 1:**
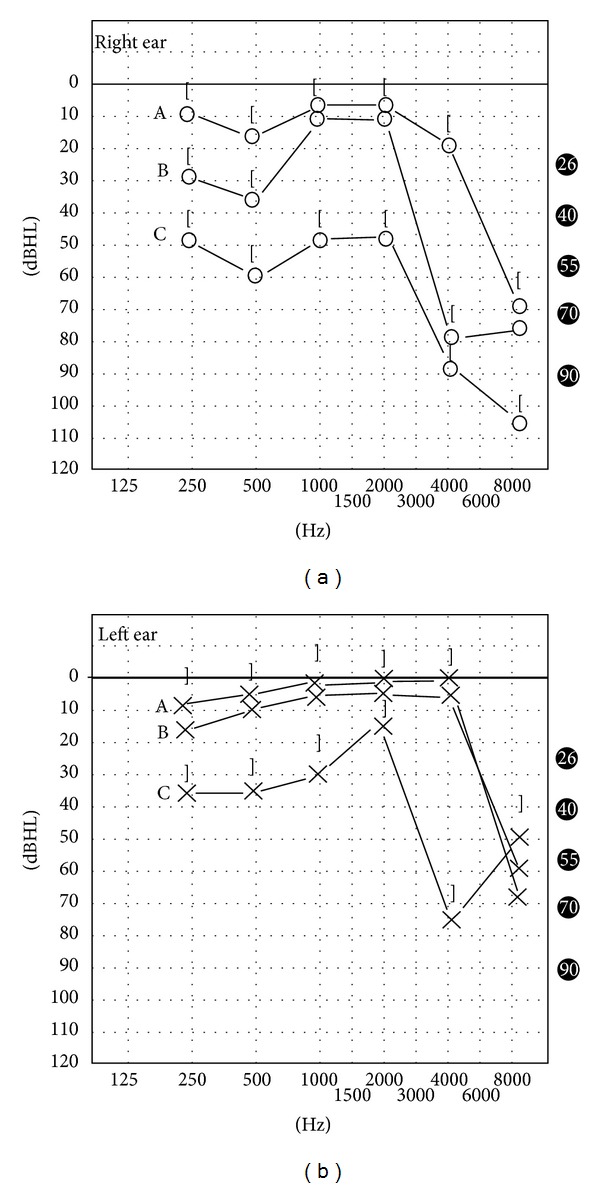
The patient's pure tone audiograms (A) on admission, (B) one month, and (C) one year after the initiation of treatment.

Upon admission, otoneurological examination revealed horizontal nystagmus with fast phase to the left. Otoscopy was normal. The tonic audiogram showed bilateral sensorineural hearing loss, greater in the right ear (Figure 1(A)); in the right ear the hearing loss was moderate with deterioration at high frequencies; in the left ear mild hearing loss was present also with deterioration at high frequencies. The patient was administered intravenous corticosteroid and vasodilator drugs. Laboratory/immunological investigation showed normal C3, C4 complement levels, raised levels of circulating immune complexes (5.4 mgEq/mL, ref. values < 4), high serum IgE immunoglobulin levels (560 IU/mL, ref. values 10–100), and normal serum IgA, IgM, and IgG immunoglobulins. Thyroid function tests (FT3, FT4, and TSH) and antithyroid peroxidase autoantibodies (anti-TPO) were normal while antithyroglobulin autoantibodies (anti-TG) were found elevated (240 IU/mL, ref. values 0–115). Protein electrophoresis was without anomalous fractions. Blood tests for autoimmunity such as FTA for syphilis, HbA1c for diabetes, HBsAg, and anti-HCV and -HIV were negative. Whole blood count, ferritin, ESR, CRP, ANA, Rf, c-ANCA, p-ANCA, anticardiolipin G,M antibodies were normal. Full blood count coagulation screening (fibrinogen, ATIII, APCR, lupus anticoagulant, and PT, aPTT) was normal.

Two days after the initiation of treatment the vertigo resolved completely. However, the patient complained that she had no improvement of hearing in the right ear, whereas she reported improvement in the left ear.

On the tenth day of treatment, electronystagmography revealed horizontal nystagmus towards the left and down only with the patient's eyes closed; with the eyes being opened this nystagmus disappeared. The caloric test showed mild (18%) unilateral right paresis and a 100% directional preponderance to the left. VEMPs were present only on the left side.

One month later, at discharge, the patient reported improvement in the left ear although with an intermittent sensation of fullness in the right ear. However, the tonic audiogram (Figure 1(B)) showed improvement on both sides, at nearly all frequencies.

Our patient was typed for HLA-A*, -B*, -C*, and -DRB1* by means of a commercially available kit (Dynal Invitrogen Corporation) using the polymerase chain reaction amplification sequence-specific primers method. HLA typing revealed A*01/24, B*08/44, Cw*04/07, and DRB1*03/11 alleles. Methylprednisolone was administered at 64 mg/day for one month, followed by gradual tapering dose in a five-month period. Four months after the initiation of the steroid treatment and on the basis of the immunologic workup at the clinical immunology section, the patient started treatment with cyclosporine-A 175 mg/day in association with methylprednisolone 16 mg/day.

Bimonthly clinical and laboratory followup, introduced to detect any cyclosporine-A side effects, revealed normal renal and hepatic function and absence of gastrointestinal side effects. After a five-month combined treatment with cyclosporine-A and methylprednisolone, clinical improvement associated with the absence of inflammation, normalization of anti-TG antibodies, and the absence of circulating immune complexes allowed steroid tapering (down to 16 mg/day). Cyclosporine-A serum concentration levels allowed further cyclosporine-A dose reduction (125 mg/day).

One year after the initiation of treatment the patient had no complains. Tonic audiogram (Figure 1(C)) shows normal hearing thresholds on both sides at all frequencies except 8 KHz where there is a fall at 70 dB.

## 3. Discussion

Primary function of HLA molecules is the participation in antigen presentation leading to T cell activation and B cell antibody production to clear infectious agents and malignant self-tissue and prevent autoimmunity by negative selection of autoreactive T cells. Population studies have shown that predisposition to almost all human autoimmune diseases is linked to HLA genes, primarily the class II genes, playing key role in the specific immune response and in parallel associated with several autoimmune diseases.

Three HLA class II haplotypes stand out as the most autoimmune-prone genetic factors: HLA-DQ2/DR3, HLADQ6/DR2, and HLA-DQ8/DR4. These three haplotypes account for almost 90% of all autoimmune diseases, while at the same time in Caucasian population studies they present with the highest frequency, suggesting that they probably have been critical for the survival of the species.

The most frequent of these haplotypes is the HLA A1-B8-DR3, found at a frequency of about 7% in European Caucasians and is associated with several autoimmune diseases including type 1 diabetes, rheumatoid arthritis, and autoimmune thyroiditis [2]. The above superhaplotype appears also to influence several aspects of the immune response by altering the balance of cytokines produced [3]. According to other investigators, the most significant imbalance concerns type 1 T helper cell responses, which are decreased in contrast to type 2 responses. Specifically, the type 2 T helper profile plays an important role in autoimmune manifestations in carriers by an increased spontaneous apoptosis of blood lymphocytes and an increased production of some autoantibodies [4]. Other studies on A1-B8-DR3 carriers indicate lower IgG (IgG2) serum levels that confers a slower clearance of the infectious agent and hence a persisting presence of it. This might allow a prolonged production of autoantibodies and a higher risk of cross-reactions [5].

So far, the only study in which the HLA A1-B8-DR3 haplotype was attempted to be related to inner ear diseases was that of Bernstein et al. [6]; in this study, 14% of 111 patients suffering from inner ear diseases such as Ménière's disease expressed the HLA A1-B8-DR3 haplotype in contrast to only 7% of the general population; they concluded [6] that these HLA genes may be the cause of an abnormal mechanism of the immune system that may result in the development of autoantibodies, circulating immune complexes, decrease in immune complex clearance, and depressed T cell function which finally affect the inner ear.

According to several studies [7, 8], HLA class II haplotypes such as HLA-DRB1 and -DQB1 have been found to be significantly increased in patients with SSNHL. Moreover, the presence of HLA class II alleles has been used as a genetic marker in the prognosis in patients suffering from SSNHL; the HLA-DQA1*01 and -DQB1*06 alleles forecast a good prognosis in patients with SSNHL; on the contrary, the HLA-DRB1*14, -DQA1*03, and -DQA1*05 alleles are associated with a poor recovery from SSNHL [9]. Finally, the HLA-Cw*07 class I allele was found to be closely associated with Ménière's disease [10, 11].

Our study showed for the first time the association of SSHL with the HLA A1-B8-DR3 haplotype, supporting the immunologic theory for the pathogenesis of SSHL. It has already been reported that SSHL can be the presenting symptom of a systemic autoimmune disease, such as Wegener's granulomatosis, relapsing polychondritis, Sjogren's syndrome, polyarteritis nodosa, systemic lupus erythematosus, or Behçet's disease [12]. Sudden sensorineural hearing loss should be distinguished from rapidly progressive bilateral hearing loss which occurs in a course of more than 3 days and within 3 months [13]; this is also an autoimmune-mediated inner ear disease which responds successfully to steroid treatment and is caused by autoantibodies against endogenous antigens causing damage to audiovestibular tissues [13].

As bilateral SSHL could be of autoimmune origin, a treatment based on steroids is recommended as a standard therapeutic procedure for a period of at least 4 weeks followed by a second phase treatment of 18 weeks with tapering doses [14]. It is important to point out the participation of corticosteroids in emergency therapeutics. Receptors of corticosteroids in cochlear and vestibular tissues of animal models have been identified, which have led to the treatment of human autoimmune diseases with intratympanic or systemic corticosteroids [15]. High levels of C3, C4, and C1q have been reported in patients with SSHL, which may identify patients that respond to anti-inflammatory drugs of the corticosteroid type [16]. The main action of corticosteroids in SSHL would be the blockage in the production of antibodies, of interleukins (IL-2, IL-3, IL-4, IL-5, IL-6), of TNF-*α* and of IFN-*γ* and the reduction of the lymphoproliferative process [16].

Cyclosporine-A, by inhibiting T lymphocyte synthesis of various cytokines (in particular IL-2, IL-4) and inducing a reduction in the CD40 ligand expression, is responsible for the inhibition of the T and B cell interaction and a further reduction in antibody production [17]. Introduction of cyclosporine-A to steroid treatment for a 5-month-course could have, as in our case suffering from bilateral SSHL, a long-term beneficial effect with clinical improvement associated with the absence of inflammation, the normalization of autoantibodies, and the absence of immune complexes that could be maintained with a minimum steroid and cyclosporine-A dose (for our case 12 mg/day, and 125 mg/day resp.).
